# Healthcare resource utilization and costs associated with renal, bone and cardiovascular comorbidities among persons living with HIV compared to the general population in Quebec, Canada

**DOI:** 10.1371/journal.pone.0262645

**Published:** 2022-07-11

**Authors:** Véronique Baribeau, Connie J. Kim, René-Pierre Lorgeoux, Josée Brisebois, Harout Tossonian, Jean Lachaine

**Affiliations:** 1 PeriPharm Inc, Montreal, Quebec, Canada; 2 Gilead Sciences Canada, Inc, Mississauga, Ontario, Canada; 3 Faculty of Pharmacy, Université de Montréal, Montreal, Quebec, Canada; University of Cape Town Faculty of Science, SOUTH AFRICA

## Abstract

There is limited understanding on healthcare utilization and costs of age-related comorbidities such as cardiovascular, bone and renal disease/disorder in people living with human immunodeficiency virus, so we compared comorbidity prevalence and associated healthcare utilization and costs. Through the Quebec health insurance database, people living with human immunodeficiency virus on antiretroviral therapy for ≥6 months from January 2006 to June 2012 were categorized by their comorbidity status using International Classification of Diseases (ICD)-9 codes, and controls without human immunodeficiency virus diagnosis or antiretroviral therapy use were age and gender matched. We compared healthcare utilization and costs. A total of 3,905 people living with human immunodeficiency virus and 11,715 control individuals were included. The mean age of people living with human immunodeficiency virus was 45.3 years and 77.3% were men. Prevalence of comorbidities was higher and occurred earlier in people living with human immunodeficiency virus and increased with older age regardless of human immunodeficiency virus status. Interestingly, bone comorbidity was high (37%) and 5-fold greater in people living with human immunodeficiency virus <20 years than the controls. Polypharmacy and comorbidity scores were greater in people living with human immunodeficiency virus than controls (p<0.01), as were cardiovascular, bone and renal comorbidities (40.3%, 26.0% and 5.5%, respectively; p<0.01). People living with human immunodeficiency virus had higher healthcare utilization and costs than controls largely due to longer hospital stays and prescriptions. Mean total healthcare cost/person/year for people living with human immunodeficiency virus was CAD$6,248 and was highest for those with renal disease (CAD$19,617). Comorbidities in people living with human immunodeficiency virus are more prevalent, occur earlier and incur a higher burden on the healthcare system; earlier screening and improved preventative and management strategies may reduce the burden to people living with human immunodeficiency virus and to the healthcare system.

## Introduction

With the widespread use of combination antiretroviral therapy (ART), HIV is mostly a manageable chronic infection and people living with HIV (PLHIV) are living longer [1, 2]. Causes of morbidity and mortality for PLHIV in Canada now mainly include non-infectious comorbidities like cardiovascular (CV) disease, hypertension, bone fractures, chronic kidney disease, liver disease, diabetes mellitus and non-AIDS-defining cancers [3–8]. Even with durable ART-induced viral control, rates of CV, bone and renal disease/disorder in PLHIV are higher than the general population [9–16]. Moreover, the presence of multimorbidity is more common in PLHIV and occurs 10–15 years earlier [15]. Factors at play likely include an excess of traditional and behavioral risk factors, the proinflammatory effect of persistent HIV and immune changes, potential ART-related toxicities on different organ systems, and disparities in healthcare access [15, 17, 18]. There is a limited number of studies that have examined the burden of non-infectious comorbidities in ART-treated PLHIV to the healthcare system, especially in Canada and those including younger PLHIV [19–26].

In order to understand the impact of comorbidities on the utilization and cost of healthcare in PLHIV, we evaluated the prevalence, healthcare resource utilization and costs associated with comorbidities (excluding ART-related costs) in PLHIV.

## Methods

### Study design and cohort selection

We performed a retrospective, cohort study in Quebec, Canada, using the Quebec public provincial health plan administrative database (*Régie de l’assurance maladie du Québec* [RAMQ]). Prescription medications in Quebec are covered by the RAMQ or private insurance. Participants in the RAMQ drug insurance program include all people who do not have access to a private medication insurance plan, beneficiaries of the social assistance program, or individuals aged 65 years and over. In addition, the RAMQ covers all physician services and hospitalizations for the entire population of Quebec.

Data on cohort characteristics and medical/pharmaceutical services were obtained from the RAMQ database and individuals were included if they were covered by the RAMQ insurance for at least 2 years. The data received from the RAMQ are fully anonymized and are based on claims from physicians and pharmacists. There is an identification number that allows researchers to link together data of each patient, but there is no way a researcher can identify a patient. The researchers define the cohort of patients and the time period for which they require RAMQ data and submit the request to RAMQ. Additional information is provided alongside the request, including study objectives, sponsors and acceptance to pay the fees for data extraction. Four to six months following the approval of the request, RAMQ provides researchers with a sample of patients from the selected cohort.

Review boards linked to the Ministry of Health agreed to deliver the dataset. All procedures were in accordance with the ethical standards of the Québec Health Insurance Board (Régie de l’assurance maladie du Québec, RAMQ). The RAMQ is a government agency that is responsible for the coding and anonymization of patient data, and already provides strict control to deliver anonymous data to researchers for specific research questions. As such, the RAMQ is inherently responsible for ethics and confidentiality. Therefore, ethical approval is considered to be granted upon the provision of their data. In this context in Québec, an approval from any other ethical board was not necessary. The data obtained from the RAMQ include an encrypted patient identifier, which enables linkage of individual patient information while preserving anonymity.

The PLHIV cohort included individuals with an HIV diagnosis (International Classification of Diseases, 9th Revision [ICD-9] codes 42.0 to 44.9) and/or with ≥1 prescription for ART between January 1, 2006 and June 30, 2012 for at least 6 months. Individuals were excluded if they were only prescribed emtricitabine/tenofovir or abacavir/lamivudine. The follow-up period ended when individuals were no longer covered by the RAMQ Drug Insurance Plan or reached the end of the study period (i.e. June 30, 2012), whichever came first.

The control cohort consisted of individuals included in the RAMQ Drug Plan by having at least one diagnosis or prescription recorded between January 1, 2006 and October 31, 2015. These individuals, without an HIV diagnosis or prescribed ART, were randomly selected. Cohort entry was defined as the date of first prescription recorded in the database.

### Matching and analyses

For every HIV case, up to three randomly selected control cases matched for age group (<20, 20–49, 50–65, >65 years) and gender were included. Several analyses were performed: 1) when comparing comorbidities, cases within PLHIV and control groups who had documented comorbidities were compared; 2) comparisons that included ≥1 comorbidity, cases included PLHIV or controls with ≥1 bone, renal or CV comorbidity; 3) finally, comparisons amongst the PLHIV group with and without comorbidity (cases of PLHIV with at ≥1 comorbidity and PLHIV without any CV, bone and renal comorbidity) were further matched by time of follow-up.

### Comorbidities identification

Cardiovascular-, bone-, and renal-related comorbidities were identified using: 1) ICD-9 codes; 2) a comorbidity-related medication; or 3) a comorbidity-related medical procedure S1 Table. Renal-related medications were not used to identify renal comorbidity since these medications are not specific for renal conditions. The comorbidity date was defined by the date of the first medication, the first diagnosis, or the first medical procedure related to the comorbidity in the 2 years following cohort entry. Comorbidity categories were not mutually exclusive.

### Comorbidity indexes

Comorbidity scores were estimated using two validated indexes, Charlson and Von Korff. The Charlson comorbidity index measures the risk of death attributable to comorbid conditions based on the individuals’ medical records, in the year after cohort entry [27]. The Von Korff comorbidity index scores the comorbidity burden according to the medications they had been prescribed and predicts hospitalization and mortality in the following year [28].

### Healthcare resource utilization and cost

Values for resource utilization and cost were reported as per person/year in Canadian dollars (CAD) and did not adjust for inflation. General healthcare resources included medical services such as hospitalization, hospitalization days, intensive care unit [ICU] visits, ICU days, emergency department [ED] visits, outpatient visits and prescription drugs (excluding ART). Health care resources related to CV, bone and renal comorbidities included consultations with specialists, medical procedures and prescribed medications. Costs for hospitalization, ICU, ED and outpatient visits were estimated using the 2012 average daily cost from the *Direction de la Gestion Intégrée de l’Information du Ministère de la Santé et des Services Sociaux* [29] (CAD$984, CAD$1,090.10, CAD$161.38, and CAD$29.31, respectively), in addition to the medical act cost taken directly from the RAMQ database.

### Statistical analysis

Demographics, clinical characteristics, comorbidity prevalence by age groups, and healthcare service utilization and costs in the 2 years following cohort entry were compared for cases and controls. Continuous variables were analyzed using mean and standard deviation (SD), and categorical variables were presented by absolute and relative frequency distributions. Cases and controls were compared with independent t-tests for continuous variables and Chi-square tests for categorical variables. Statistical analysis were performed using ‘IBM SPSS Statistics for Windows, version 19 (IBM Corp., Armonk, N.Y., USA).

## Results

### Characteristics and comorbidity prevalence in PLHIV and matched controls

From the RAMQ database, 3,905 PLHIV and 11,715 control individuals without a record of HIV diagnosis or ART prescription were included (S1 Fig). Most individuals were male (77.3%) and the mean age of PLHIV and controls groups were 45.3 and 44.7 years, respectively (Table 1). Both the Charlson and Von Korff comorbidity index scores were higher in PLHIV than controls Table 1, p<0.01, as was polypharmacy (10.3 vs. 5.2 different drugs used, p<0.01). More PLHIV had at least one comorbidity (53.6% vs. 43.8%, p<0.01), and there were over 2.5-fold more individuals with two or more categories of comorbidities in the HIV group compared to the control group (17.1% vs. 6.5%, p<0.01). Consistent with these results, CV, bone and renal comorbidities were all more frequent in PLHIV than matched controls (40.3% vs. 36.4%, 26.0% vs. 11.3%, and 5.5% vs. 1.5%, respectively, p<0.01).

**Table 1:** Demographics and clinical characteristics of PLHIV and controls.

Demographic Characteristics	PLHIV (n = 3,905)	Control^a^ (n = 11,715)	p Value^b^
**Male, n (%)**	3,020 (77.3)	9,060 (77.3)	1.00
**Mean age, years (SD)**	45.3 (11.9)	44.7 (13.7)	0.01
**Age Groups, years, n (%)**			
<20	67 (1.7)	201 (1.7)	1.00
20–49	2,542 (65.1)	7,626 (65.1)	1.00
50–65	1,057 (27.1)	3,171 (27.1)	1.00
>65	239 (6.1)	717 (6.1)	1.00
**Comorbidity Scores and Polypharmacy**			
Charlson comorbidity score, mean (SD)	3.3 (3.3)	0.3 (0.8)	≤0.01
Von Korff comorbidity score, mean (SD)	2.7 (3.0)	1.9 (2.6)	≤0.01
Number of different drugs used, mean (SD)	10.3 (8.1)	5.2 (5.3)	≤0.01
**Comorbidity Prevalence**			
No comorbidity, n (%)	1,810 (46.4)	6,579 (56.2)	≤0.01
≥1 comorbidity, n (%)	2,095 (53.6)	5,136 (43.8)	≤0.01
≥2 comorbidities, n (%)	667 (17.1)	763 (6.5)	≤0.01
Any CV,^c^ n (%)	1,574 (40.3)	4,263 (36.4)	≤0.01
Any Bone,^c^ n (%)	1,014 (26.0)	1,323 (11.3)	≤0.01
Any Renal,^c^ n (%)	213 (5.5)	180 (1.5)	≤0.01

ART: antiretroviral therapy; CV: cardiovascular disease; SD: standard deviation.

^a^Controls were matched for age group and gender to PLHIV.

^b^p values for the comparison of PLHIV to the matched control group are from chi-square tests for categorical variables and independent t-tests for continuous variables.

^c^Comorbidity categories are not mutually exclusive since one patient could have more than one type of comorbidity.

### Comorbidity burden by age group in PLHIV and matched controls

**Fig 1:**
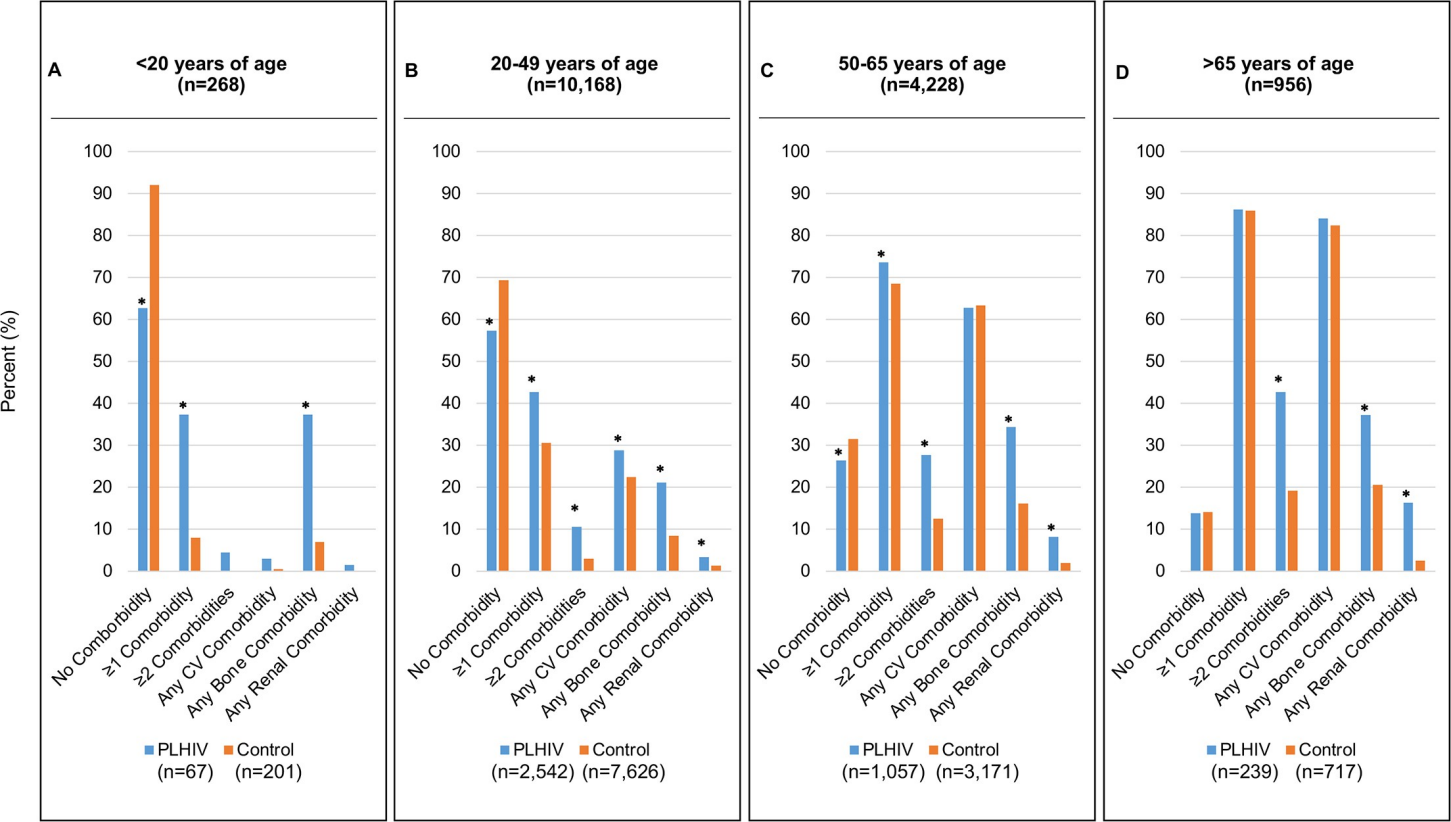
Distribution of comorbidities by type for different age groups of PLHIV and controls. (A) <20 years of age, (B) 20–49 years of age, (C) 50–65 years of age, (D) >65 years of age.

The prevalence of comorbidities increased with age category, regardless of HIV status (Fig 1). In all younger individuals (<20 years), bone disorders, including osteoporosis and fractures, were the most common comorbidity and was over 5-fold more prevalent in PLHIV than matched controls (37.3% vs. 7.0%, p<0.01) (Fig 1A). In those between the ages of 20 and 49, PLHIV had higher cardiovascular, bone, renal, or at least one or two comorbidity categories compared to matched controls (p<0.01 for all) (Fig 1B). In particular, PLHIV had 3.5-fold higher prevalence of two or more co-existing comorbidity categories than controls (10.6% vs. 3.0%, p<0.01). In the older age groups (50–65 years and >65 years), bone, renal and/or having at least two comorbidities were more prevalent in PLHIV (p<0.01 for all) (Fig 1C and 1D).

### Healthcare utilization in PLHIV and matched controls by age

Overall, healthcare utilization in the 2 years following cohort entry for PLHIV was greater for all medical services and prescription drugs compared to controls (Table 2). PLHIV used 2.0-fold more medical services (sum of hospitalization days, ICU days, ED and outpatient visits; 10.6 vs. 5.3 days, p<0.01) and had 2.7-fold more mean annual prescription drugs than matched controls (70.5 vs. 26.6 drugs/person/year, p<0.01) for all ages. Outpatient visits was the largest contributor to medical services (9.1 visits/person/year), followed by hospitalization days, ED visits and ICU days (2.2, 1.0 and 0.2 visits/person/year respectively). Number of prescription drugs excluding ART was also 2.7-fold higher in PLHIV (70.5 vs 26.6 drugs/person/year) in all age comparison. These trends were seen for all age cohorts with some variability in the <20-year group.

**Table 2:** Healthcare utilization in the 2 years following cohort entry for PLHIV in different age groups compared to controls.

	All Ages		<20 years old		20–49 years old		50–65 years old		>65 years old	
Healthcare Utilization	PLHIV	Controls	PLHIV	Controls		Controls	PLHIV	Controls	PLHIV	Controls
n	3,905	11,715	67	201	2,542	7,626	1,057	3,171	239	717
**All Medical Services**† Mean (SD)/Median	10.6* **(12.5)** 8.0	5.3 (6.5) 3.5	7.8* **(5.8)** **6.5**	3.9 (4.8) 2.5	9.6* **(11.1)** **7.5**	4.8 (6.4) 3.0	11.7* **(13.2)** **9.0**	5.9 (6.8) 4.5	16.3* **(20.4)** **12.0**	8.0 (5.8) 6.5
Outpatient visits, mean #/person/yr (SD)/median	9.1* **(11.5)** 7.0	4.4 (5.8) 3.0	6.6* **(4.6)** **5.5**	3.3 (4.6) 2.0	8.1* **(10.1)** **6.5**	3.9 (5.6) 2.5	10.3* **(12.3)** **8.0**	5.2 (6.2) 4.0	14.5* **(19.7)** **0.0**	7.1 (5.2) 0.0
Hospitalization days, mean # days/person/yr (SD)/median	2.2* **(8.2)** **0.0**	0.9 (6.5) 0.0	2.1 (9.2) 0.0	0.1 (0.5) 0.0	1.9* **(6.9)** **0.0**	1.0 (7.7) 0.0	2.8* **(10.8)** **0.0**	0.8 (3.6) 0.0	3.0* **(8.0)** **0.0**	0.9 (3.5) 0.0
ED visits, mean #/person/yr (SD)/median	1.0* **(2.4)** **0.0**	0.6 (1.4) 0.0	0.6 (1.1) 0.0	0.5 (1.0) 0.0	1.1* **(2.6)** **0.0**	0.7 (1.6) 0.0	0.9* **(1.8)** **0.0**	0.6 (1.1) 0.0	1.0* **(2.2)** **0.0**	0.6 (1.1) 0.0
ICU days, mean # days/person/yr (SD)/median	0.2* **(1.2)** **0.0**	0.1 (0.6) 0.0	0.1 (0.5) 0.0	<0.1 (0.01) 0.0	0.1* **(0.7)** **0.0**	<0.1 (0.5) 0.0	0.2* **(1.7)** **0.0**	0.1 (0.7) 0.0	0.3 (1.9) 0.0	0.1 (0.5) 0.0
**Prescription drugs, mean #/person/yr (SD)/median**	70.5* **(154.6)** **25.5**	26.6 (71.0) 6.0	19.3* **(44.1)** **0.0**	5.2 (7.5) 0.0	66.7* **(168.7)** **0.0**	21.2 (75.1) 0.0	80.4* **(131.3)** **0.0**	37.0 (64.3) 0.0	80.5* **(97.6)** **0.0**	44.8 (53.5) 0.0

#: number; ED: emergency department; ICU: intensive care unit; pt: patient; SD: standard deviation; yr: year.

*p≤0.01 for the comparison of PLHIV and matched controls from independent t-tests for continuous variables; ^†^Includes number of hospitalizations and outpatient, ED and ICU visits.

### Healthcare utilization by comorbidity status and age in PLHIV and matched controls

To compare healthcare utilization between PLHIV and controls by specific comorbidity prevalence, we compared medical services and prescription drug use in the two groups by matching for CV, bone and renal comorbidity status and age. Indeed, PLHIV with any CV, bone and renal disease/disorder had greater healthcare utilization (p<0.01 for all comparisons) S2 Table. In the <20 years old group, PLHIV having any bone disorder had greater healthcare utilization (S3 Table), which was consistently observed in all older cohorts with bone comorbidity. Similar trends were seen in individuals with CV comorbidity compared to matched controls across all groups, and these differences were significant in the 20–49 and older groups (S4 Table, p<0.01). Trends for increased medical service use in PLHIV with any renal comorbidity compared to matched controls were seen across all age groups but were only significant for prescription drugs in the 20–49 and 50-65-year-old cohorts (S5 Table, p<0.01).

### Healthcare costs in PLHIV and matched controls

**Fig 2:**
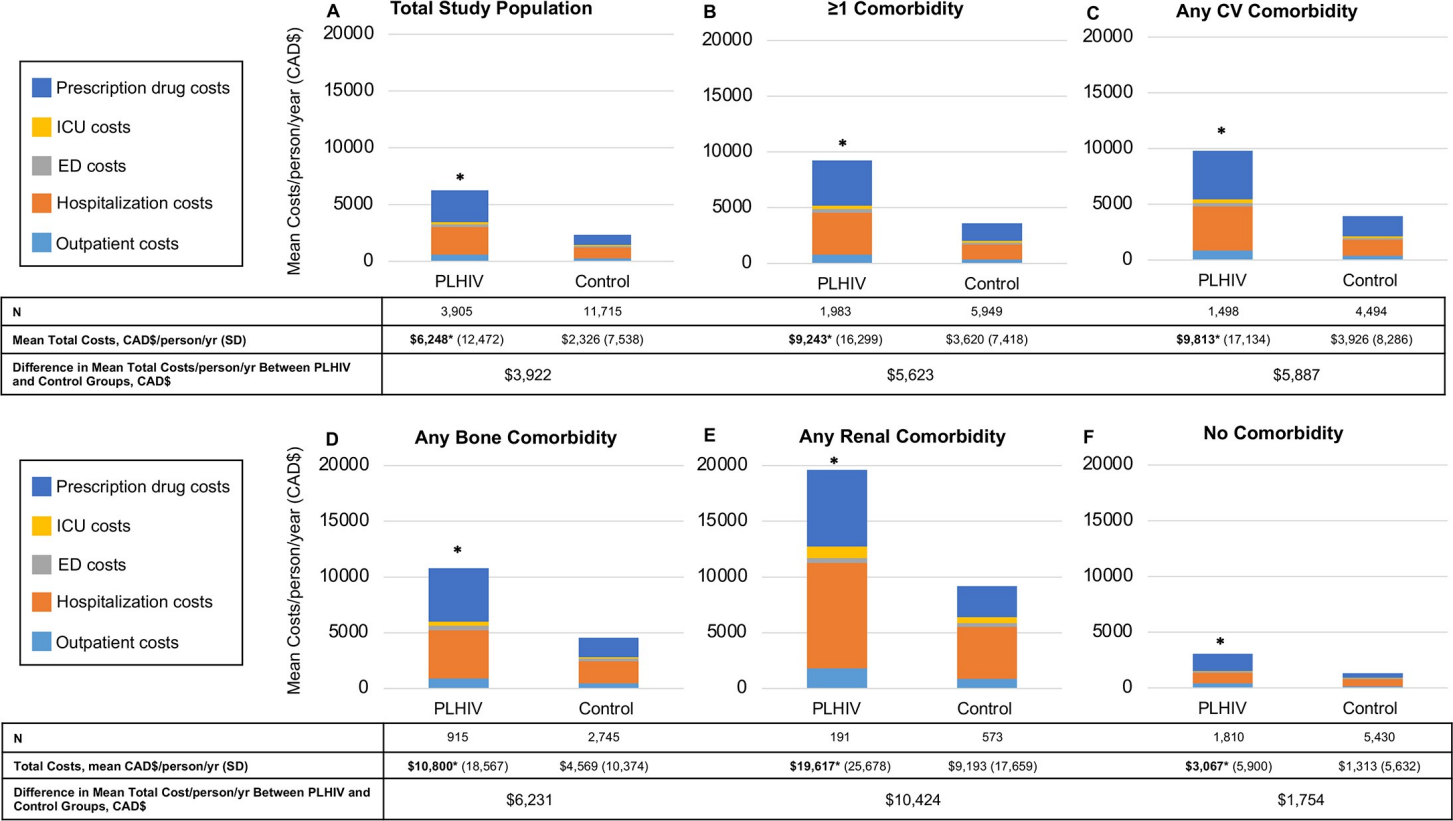
Healthcare costs among PLHIV and controls by comorbidity status. Mean healthcare costs per person per year are shown in the top row for PLHIV and matched control individuals, for those with: (A) all individuals, (B) at least one comorbidity, (C) any CV comorbidity, (D) any bone comorbidity, (E) any renal comorbidity, and (F) no CV, bone or renal comorbidities. Comorbidity categories are not mutually exclusive since an individual could have more than one type of comorbidity.

Similarly, total healthcare costs (sum of ICU, ED, hospitalization, outpatient care and drug costs) for PLHIV were at least 2-fold higher compared to controls regardless of comorbidity status Fig 2 p<0.01 for all. Prescription drug (excluding ART) and hospitalization costs made up the bulk of total costs in similar amounts for persons with any bone or CV disease (hospitalization: 41%; drugs: 44%), whereas the almost half of costs for those with renal comorbidity were due to hospitalization (hospitalization: 48%; drugs: 35%). For all individuals, the mean total healthcare cost for PLHIV was higher than controls by CAD$3,922/person/year Fig 2A, as were the individual cost components (drugs, hospitalization, ICU, ED, and outpatient visits) (p<0.01). Similarly, PLHIV with at least one comorbidity, with any CV, bone or renal comorbidity, and without any comorbidity Fig 2B–2F, had higher healthcare costs by CAD$5,623, CAD$5,887, CAD$6,231, CAD$10,424 and CAD$1,754/person/year, respectively, compared to matched controls (p<0.01 for all comparisons).

### Healthcare utilization and costs in PLHIV with and without comorbidity

To better elucidate the healthcare utilization and costs of having any comorbidity in PLHIV, we performed matched analyses of PLHIV with at least 1 comorbidity vs. those without S6 Table. Mean total healthcare costs for PLHIV with at least 1 comorbidity were 2.7-fold greater than matched PLHIV without comorbidity (CAD$9,184 vs. CAD$3,366 per person per year, p<0.01). Likewise, all medical services utilization and prescription drug usage were greater in PLHIV with comorbidities than without (p<0.01).

### Total healthcare costs of PLHIV by comorbidity status

**Fig 3:**
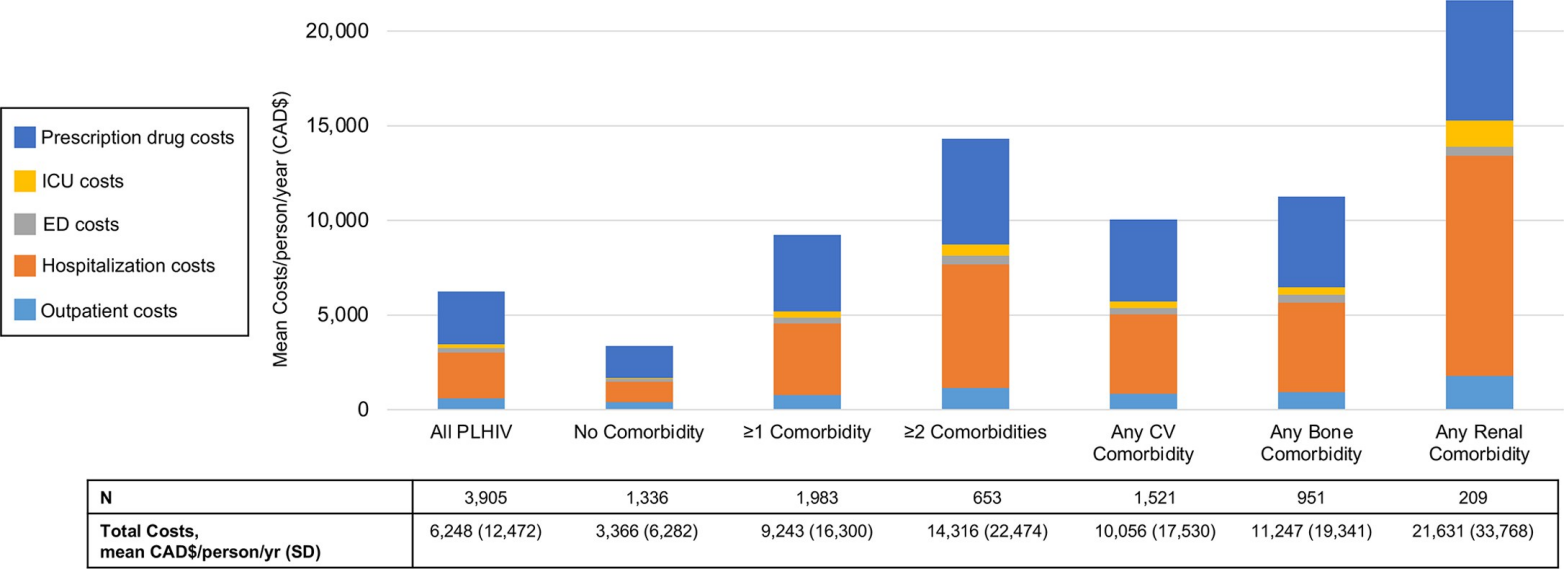
Mean total healthcare costs of PLHIV without CV, bone or renal comorbidity and PLHIV in different comorbidity categories. Mean healthcare costs per patient per year are shown for all patients, as well as those with at least one comorbidity, any CV comorbidity, any bone comorbidity, any renal comorbidity, and no CV, bone or renal comorbidities. Comorbidity categories are not mutually exclusive since one patient could have more than one type of comorbidity.

The overall mean total healthcare cost (excluding ART) of all PLHIV was CAD$6,248/person/year Fig 3. Individuals without CV, bone or renal comorbidity incurred CAD$3,366/person/year, and on average total costs increased by approximately CAD$5,000/person/year with increasing levels of comorbidity (CAD$9,243 for PLHIV with at least 1 comorbidity and CAD$14,316 for PLHIV with at least 2 comorbidities). The highest total costs were seen in PLHIV with any renal comorbidity (CAD$21,631/person/year); the largest contributor was hospitalization (CAD$11,614), followed by prescription drugs (CAD$6,360).

## Discussion

Even though the life expectancy of PLHIV is comparable to that of the general population in the modern ART era, earlier onset and higher prevalence of comorbidities negatively affect the quality of life experienced by PLHIV. We have limited understanding of the economic burden of this disparity as few studies have investigated comorbidity-related healthcare utilization [19–21]. Comorbidities in PLHIV appear to markedly increase the cost of inpatient and outpatient care but appear to have a minimal impact on drug costs, which are dominated by ART [19, 22–24]. In this retrospective real-world cohort study, PLHIV had a higher prevalence of CV, bone, and renal comorbidities than their age and gender matched peers regardless of age group. Importantly, bone disorders were prevalent in over a third of PLHIV <20 years of age and prevalence was 5-fold greater than controls. This increased burden of comorbidities in PLHIV was associated with significant increases to healthcare utilization and costs, driven largely by outpatient visits and non-ART prescription drugs.

We showed that as PLHIV age and develop comorbidities, the annual healthcare costs increase through increased medical service and prescription drug use. Similar to our study, an Italian cohort showed that non-infectious comorbidity-related medical costs in PLHIV (hypertension, diabetes, CVD, fractures and renal failure) was US$2000/year in PLHIV <40 years old and over US$10,000 in PLHIV >60 years of age [22]. Similarly, Quiros-Roldan et al. showed per capita costs in PLHIV increased by 36€ for every year of age and costs were 3700€ more for PLHIV with at least one chronic disease compared to those without [24]. The same study also showed that the cost of treating renal failure was the highest at 13,665€/person/year, and our study also shows renal comorbidity to be the costliest at CAD$19,617/person/year. In our analysis, renal disease was the least common comorbidity for PLHIV in all age groups but was associated with the highest burden on total healthcare costs and healthcare utilization, driven largely by hospitalization costs. While advances with HIV therapy have reduced the impact of HIV infection on the development of HIV-associated nephropathy, factors like black race, female sex, older age, diabetes, hypertension, hepatitis C co-infection, and illicit drug use may increase the risk of renal comorbidities; however, we were unable to collect and adjust for these factors [30, 31]. Of particular importance is the earlier onset of renal comorbidity in PLHIV. We showed higher prevalence of renal, bone and CV comorbidity and multimorbidity in younger PLHIV (<50 years) compared to controls, consistent with previous studies [11, 15, 32, 33]. Other studies suggest that multimorbidities in PLHIV emerge 10–15 years earlier than controls [15] and large HIV cohorts such as the NA-ACCORD reported >10-fold higher rate of end-stage renal disease (ESRD) for PLHIV <50-years [31]. Healthcare costs for chronic kidney disease (CKD) and ESRD are high, with the majority of costs associated with renal replacement therapies, especially in-hospital hemodialysis [34]. In addition, earlier stages of CKD have been shown to increase healthcare costs due to other complications such as CV, endocrine, gastrointestinal, hematological and osteoarticular abnormalities [34, 35]. Minimizing risks of developing CKD in PLHIV should be prioritized and include identification of worsening of renal function, discontinuation of potentially nephrotoxic therapies, routinely measuring estimated glomerular filtration rate (eGFR) and proteinuria to identify high-risk individuals who may require treatment, and referring persons with moderate to severe CKD to a nephrologist [36].

While the renal comorbidity had the greatest healthcare utilization, the onset of bone comorbidity or disorders in PLHIV was the earliest. To our knowledge, we are the first to report non-infectious comorbidities in PLHIV increasing healthcare resource utilization in individuals <20 years old compared to controls from the general population. Having any bone comorbidity was higher in PLHIV than controls at all age groups and the healthcare cost was >2-fold greater compared to controls. We surprisingly observed nearly a third of all PLHIV <20 years of age to have a bone comorbidity/disorder and this was consistent with Guaraldi et al.’s study which showed the comparative risk of bone fracture being high across all age strata with HIV [15]. One limitation with our study was that bone comorbidity included both osteoporosis and bone fractures S1 Table and we were unable to discern traumatic fractures which may be more prevalent in younger individuals. Although we did not have data on duration of HIV infection or if these individuals acquired HIV perinatally, it is known that HIV infection reduces bone accrual throughout childhood and adolescence and reduces low peak bone mass [37]. This negative HIV-associated bone health impact may increase their healthcare utilization and costs as observed in our study [37]. In contrast to our study, a US retrospective study using administrative health claims data from individuals with commercial and public insurance showed costs for PLHIV with and without fracture/osteoporosis to be similar, although healthcare costs for PLHIV with CKD and CV disease were greater than controls [20]. However, their bone comorbidity data was limited to privately-insured persons, who may have different socioeconomic characteristics than individuals in the present study. Factors thought to play a role in reduced bone mass in PLHIV include certain antiretroviral medications (e.g. protease inhibitors and tenofovir disoproxil fumarate [TDF]), in addition to delayed biologic maturation [37–39]. Based on our results and others, more research is needed to understand how healthcare utilization and costs for young people and perinatally infected individuals with bone comorbidity (and possibly other comorbidities) differ over time compared to PLHIV with bone comorbidity infected later in adulthood. Our data also suggest healthcare utilization studies on comorbidity in PLHIV may be missing valuable information if analyses are limited to the commonly used cut-off of 40 or 50 years of age to separate younger from older PLHIV.The cause of earlier onset of comorbidities in PLHIV with controlled HIV is multifactorial and includes a combination of social, behavioral and host factors, which were not assessed as they were beyond the scope of this study. Increased immune activation and chronic inflammation are thought to play a pathogenic role in PLHIV [40] and factors such as smoking, substance abuse, hepatitis C coinfection, food insecurity or social isolation can also affect aging, healthcare access and progression or severity of comorbidities [41]. Guaraldi et al. showed that independent predictors of total healthcare costs included age, male sex, multimorbidity, and a nadir CD4 cell count of <200 cells/mm^3^, and a French study of PLHIV on ART showed higher mortality risk with increasing social vulnerability even when known clinical and behavioural predictors of mortality were controlled for [22, 42]. Social vulnerability is often associated with mental health issues like depression, a major predictor of clinical progression [43]. The greater tendency of this population to use emergency services compared to “socially secure” PLHIV represents a missed opportunity for early detection of developing comorbidities (e.g. non-AIDS-related cancers) or risk factors for acute fatal events (e.g. cardiovascular events) [44].

This study has several limitations. Younger persons or those of higher socioeconomic status are likely underrepresented in the RAMQ database, since the RAMQ drug insurance only covers people aged 65 and over, beneficiaries of the social assistance program and individuals who do not have access to a private medication insurance plan. Due to the lack of laboratory or procedure test results, HIV diagnosis could not be confirmed with the available data. However, misdiagnosis of HIV was unlikely since individuals included in the analysis were required to have an ART prescription for at least 6 months to be included. More vigilant medical care among PLHIV compared to the general population may have led to the greater level of diagnosed and reported comorbidities compared to controls. To help minimize this bias, controls had to have had at least one prescription or one diagnosis identified in the database to ensure they had at least one medical visit. These data may have underestimated the prevalence of renal comorbidities since medications were not used for identification; in addition, total costs associated with comorbidities could have been underestimated due to missing undiagnosed cases or lack of a drug prescription or ICD-9 code associated with some individuals. Unmeasured confounding (e.g. behavioral risk factors) was not accounted for in the matching analyses. Because the observation period covered January 2006 to June 2012 only, comorbidities and HIV management data are older and care may have changed and/or improved since that time. A final limitation is that costs reported were not adjusted for inflation.

Our study contributes to the growing body of evidence on the greater burden and earlier emergence of comorbidities in PLHIV on ART, and the associated increase in healthcare resource utilization and cost. Many of the comorbidities observed in PLHIV can be addressed through currently available prevention measures directed at curbing traditional risk factors [19, 31]. Earlier screening of traditional risk factors and comorbidities, appropriate ART selection that reduce risk of certain comorbidities, along with the use of interventions that have been tailored to better address the needs of PLHIV. A holistic care model that integrates the best medical interventions and treatments and focuses on empowerment and wellbeing of the individual person living with HIV can improve the long-term health of PLHIV.

## Supporting information

S1 Fig Flowchart of selection of PLHIV and control patients without a record of HIV diagnosis or ART. (PDF)

S1 Table ICD-9 codes, medications and procedures used to identify comorbidities. (PDF)

S2 Table Health care utilization by age group. (PDF)

S3 Table Health care services utilization and costs for HIV-positive patients with bone comorbidity and for a matched control group of HIV-negative patients with bone comorbidity by age group. (PDF)

S4 Table Health care services utilization and costs for HIV-positive patients with CV comorbidity and for a matched control group of HIV-negative patients with CV comorbidity by age group. (PDF)

S5 Table Health care services utilization and costs for HIV-positive patients with renal comorbidity and for a matched control group of HIV-negative patients with renal comorbidity by age group. (PDF)

S6 Table Mean total healthcare costs for PLHIV with at least 1 comorbidity vs. matched PLHIV without comorbidity. (PDF)
