# Supplementary material for: Healthcare resource utilization and costs associated with renal, bone and cardiovascular comorbidities among persons living with HIV compared to the general population in Quebec, Canada
Source: PLoS One. 2022 Jul 11;17(7):e0262645. doi: 10.1371/journal.pone.0262645 (PMC9273062; doi:10.1371/journal.pone.0262645)
Supplement: S1 Fig — (PDF) [file pone.0262645.s001.pdf]

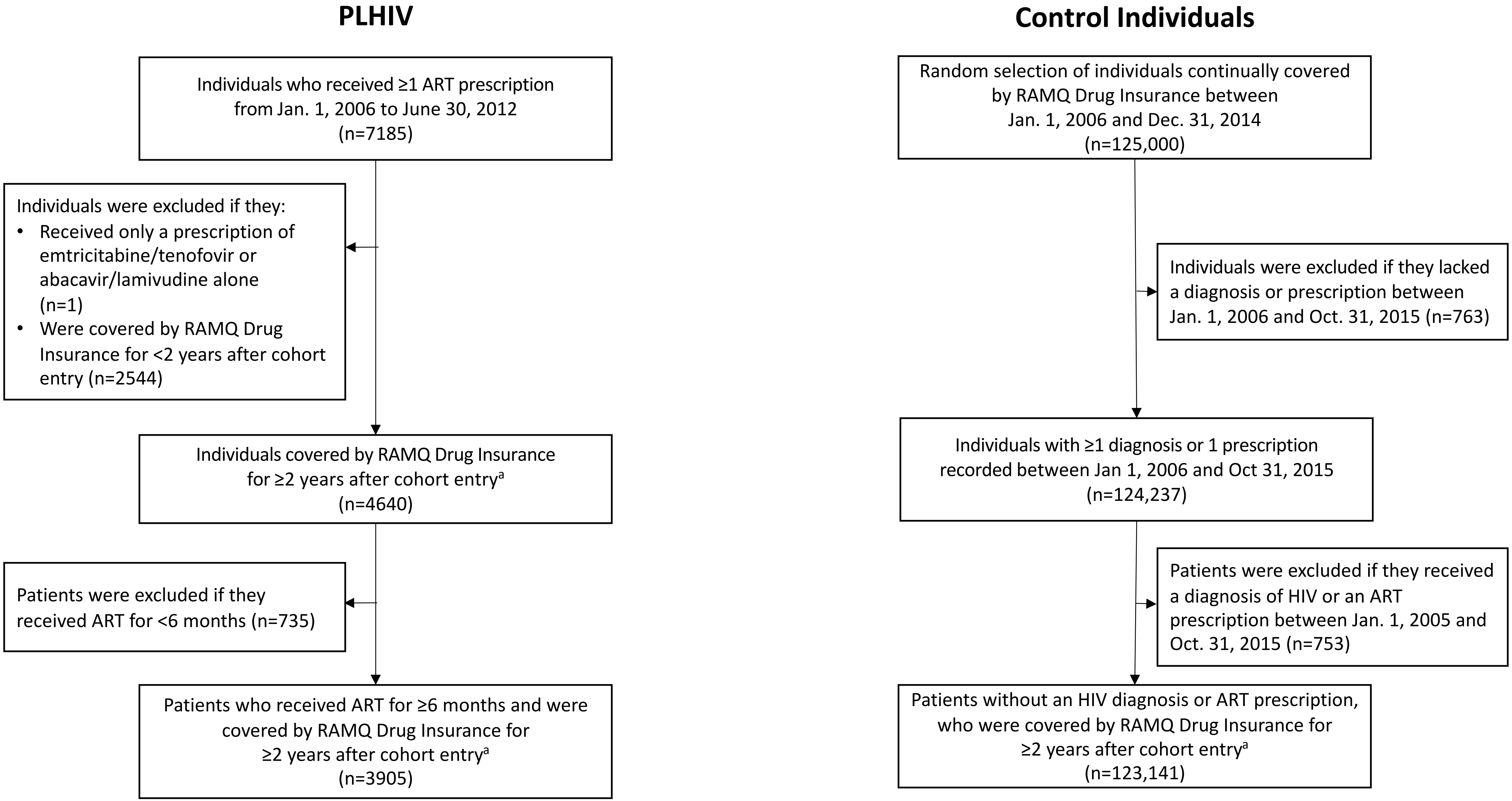

S1 Fig. Flowchart of selection of PLHIV and control patients without a record of HIV diagnosis or ART.
