# Supplementary material for: Healthcare resource utilization and costs associated with renal, bone and cardiovascular comorbidities among persons living with HIV compared to the general population in Quebec, Canada
Source: PLoS One. 2022 Jul 11;17(7):e0262645. doi: 10.1371/journal.pone.0262645 (PMC9273062; doi:10.1371/journal.pone.0262645)
Supplement: S1 Table — (PDF) [file pone.0262645.s002.pdf]

S1 Table. ICD-9 codes, medications and procedures used to identify comorbidities.

COMORBIDITIES	ICD-9 CODES
Cardiovascular	
Angina	413.x
Ischemic heart disease	410.0-412.9, 414.x
TIA	435.x
Hypertension	401.0-405.9
Cerebrovascular disease	434.X, 436.x, 437.x
Bone	
Osteoporosis	733.0
Bone fractures	800.0-829.9
Renal	
Acute glomerulonephritis	580.x
Nephrotic syndrome	581.x
Chronic glomerulonephritis	582.x
Nephritis and nephropathy	583.x
Acute renal failure	584.x
Chronic kidney disease	585.x
Renal failure	586.x
Dialysis	V45.1, V56.x
Renal transplantation	V42.0
Renal calculi	592.0
Cystic diseases of kidney	753.1
Diabetic nephropathy	250.3
Hypertensive nephropathy	403.x

ICD-9: International Classification of Diseases, 9th revision; TIA: Transient ischemic attack.

Medications considered for cardiovascular, bone and renal comorbidities

Therapeutic class	INN (RAMQ codes)
CV and hypertension medications	
ACE inhibitors	Captopril (42071), Fosinopril (47002), Enalapril (45476, 45572), Lisinopril (45576, 47040), Ramipril (47079, 46216, 47655), Perindopril (47117, 46258, 47449), Quinapril (45629, 47301), Benazepril (47049), Cilazapril (46194, 47056), Fosinopril (47002), Trandolapril (47250, 47440)
Angiotensin II receptor blockers	Losartan (47135, 46284, 46441, 47207), Candesartan (47309, 46529, 47412, 46760), Valsartan (47259, 46418, 47369), Eprosartan (47389, 47532, 47534), Irbesartan (47282, 46459, 47354), Telmisartan (47333, 46587, 47413), Olmesartan (47763, 47764)
Diuretics	Hydrochlorothiazide (4537), Bendroflumethiazide (806, 46157), Furosemide (4173), Spironolactone (9100, 46572, 38158), Chlorthiazide (1846), Amiloride (41759, 41772), Chlorthalidone (1976), Furosemide (4173), Indapamide (43397), Bumetanide (46294), Torasemide (46379), Triamterene (9763, 38197, 46772), Sodium ethacrynate (3549), Metolazone (19440), Epleronone (47766)
Beta-Blockers	Metoprolol (38275, 46763, 46780), Nadolol (40563, 46157), Atenolol (43670, 46325, 46315), Oxprenolol (42162), Nebivolol (47970), Pindolol (39016, 45408), Propranolol (8229), Timolol (38314), Acebutolol (45463), Betaxolol (45440), Bisoprolol (47355), Labetolol (45243), Carvedilol (47199, 46319)
Calcium channel blockers	Amlodipine (47006, 47609, 47889), Diltiazem (43228, 47247), Nifedipine (42708, 46388, 46469, 47751), Felodipine (45624), Nicardipine (45571), Verapamil (40550, 46573)
Renin inhibitors	Aliskerin (47706, 47823)
HMG-coenzyme A reductase inhibitors (statins)	Atorvastatin (47609, 46355, 47232), Simvastatin (45564, 46584), Lovastatin (45500, 47604), Pravastatin (45570, 47169, 47595), Rosuvastatin (46860), Fluvastatin (47083, 46240), Cerivastatin (47272)
Fibrates	Clofibrate (2067), Bezafibrate (47092), Gemfibrozil (44879), Fenofibrate (47366, 47373, 47596, 47754, 45574, 46575)
Lipid agent	Ezetimibe (47456), Dextrothyroxine (2704), Probucol (38392)
Peripheral vasodilator	Niacin (6487, 19089, 47604, 46803, 47560, 46147), Isoxsuprine (46025), Phentolamine (7449), Pentoxifylline (44346), Ergoloids (46180), Cyclandelate (46193)

Therapeutic class	INN (RAMQ codes)
Anticoagulants	Warfarin (10218, 46604, 10205), Heparin (18179, 45538, 46095, 4407, 46415, 45497), Dabigatran (47802), Rivaroxaban (47756), Dalteparine (47125, 46268), Danaparoide (47279), Enoxaparine (47026), Fondaparinux (47443), Nadroparine (47264), Tinzaparine (47163, 47098, 9919, 42864), Acenocoumarol (13)
Thrombolytics	Streptokinase (41941, 9126)
Antiplatelet agents	Aspirin (143, 46353), Clopidogrel (47307, 46486), Dipyridamole (3094), Ticlopidine (45617, 47402), Prasugrel (47834), Tirofiban (47337), Eptifibatide (47348)
Nitrates	Nitroglycerine (9919, 42864)
Bone-related medications	
Bisphosphonate	Alendronate (46295, 47165, 47747, 47662), Zoledronate (47386, 46695), Risedronate (46631, 47339, 47630), Etidronate (39796, 47142), Pamidronate (45628), Clodronate (47001, 47010)
RANKL inhibitor	Denosumab (47842)
Bone formation agent	Teriparatide (47515)
Hormonal therapy	Estrogen (34232, 45582, 45583, 47395, 43059, 43072, 6682), Raloxifene (46510, 47340), Calcitonin (40862, 46589)
Supplements of calcium and vitamin D	Calcium (1261, 1287, 1274, 1287, 1300, 1313, 1326, 1352, 1664, 5421, 18868, 40524, 44983, 45552, 46096, 46103, 46165, 46523, 46647, 46666, 46867, 47351, 47602, 47057, 47470, 47544, 47672, 47719, 47790, 47794, 47851, 47888), Vitamin D (45295, 46630), Ergocalciferol (47036), Alfacalcidol (41642), Calcitriol (40589), Cholecalciferol (45484)
Renal medications	
ACE inhibitors	Captopril (42071), Fosinopril (47002), Enalapril (45476, 45572), Lisinopril (45576, 47040), Ramipril (47079, 46216, 47655), Perindopril (47117, 46258, 47449), Quinapril (45629, 47301), Benazepril (47049), Cilazapril (46194, 47056), Fosinopril (47002), Trandolapril (47250, 47440)
Corticosteroids	Prednisone (8021)
Diuretics	Hydrochlorothiazide (4537), Bendroflumethiazide (806, 46157), Furosemide (4173), Spironolactone (9100, 46572, 38158), Chlorthiazide (1846), Amiloride (41759, 41772), Chlorthalidone (1976), Furosemide (4173), Indapamide (43397), Bumetanide (46294), Torasemide (46379), Triamterene (9763, 38197, 46772), Sodium ethacrynate (3549), Metolazone (19440), Epleronone (47766)

Therapeutic class	INN (RAMQ codes)
Beta-Blockers	Metoprolol (38275, 46763, 46780), Nadolol (40563, 46157), Atenolol (43670, 46325, 46315), Oxprenolol (42162), Nebivolol (47970), Pindolol (39016, 45408), Propranolol (8229), Timolol (38314), Acebutolol (45463), Betaxolol (45440), Bisoprolol (47355), Labetolol (45243), Carvedilol (47199, 46319)
Calcium channel blockers	Amlodipine (47006, 47609, 47889), Diltiazem (43228, 47247), Nifedipine (42708, 46388, 46469, 47751), Felodipine (45624), Nicardipine (45571), Verapamil (40550, 46573)
Angiotensin II receptor blockers	Losartan (47135, 46284, 46441, 47207), Candesartan (47309, 46529, 47412, 46760), Valsartan (47259, 46418, 47369), Eprosartan (47389, 47532, 47534), Irbesartan (47282, 46459, 47354), Telmisartan (47333, 46587, 47413), Olmesartan (47763, 47764)
Potassium lowering agents	Sodium/Calcium polystyrene sulfonate (7787, 44931, 46451)
Supplements of calcium and vitamin D	Calcium (1261, 1287, 1274, 1287, 1300, 1313, 1326, 1352, 1664, 5421, 18868, 40524, 44983, 45552, 46096, 46103, 46165, 46523, 46647, 46666, 46867, 47351, 47602, 47057, 47470, 47544, 47672, 47719, 47790, 47794, 47851, 47888), Vitamin D (45295, 46630), Ergocalciferol (47036), Alfacalcidol (41642), Calcitriol (40589), Cholecalciferol (45484)
Xanthine oxidase inhibitors	Allopurinol (195), Febuxostat (47843)

INN : international Nonproprietary Name; CV : cardiovasuclar; ACE : angiotensin-converting-enzyme.

Medical procedures considered for cardiovascular, bone and renal comorbidities

**Medical procedures**	**Codes**
Cardiac procedures	
Coronary angiography	294 and 488
Stenting	631, 632, 662 and 20521
Echocardiogram and nuclear stress test	339, 309 and 329
Electrocardiogram	340, 341 and 350
Heart CT scan and electron-beam computerized tomography	8291, 8292, 8293 and 8294
Carotid endarterectomy	4710
Bypass surgery	4601-4606
Mechanical clot removal	9446
Bone-related procedures	
Cast and bracing	2509, 2508, 2507, 2525, 9569, 2533, 18083, 2581, 18085, 18096, 2546, 2544, 2605, 2568, 2606, 2598, 2608, 2609, 2640, 2610, 2912, 2662, 18041, 18064, 2651, 18043, 18045, 18046, 18049, 18050, 2619, 2629, 2747, 2748, 18105, 18106, 2690, 2737, 2749, 2694, 2708, 2710, 2744, 2711 and 2691
Dexa scans	8243 and 8245-8247
Surgery for fractures	2512, 2520, 2521, 2515, 2513, 2514, 2522, 2526, 9570-9577, 2535, 18081, 18082, 2572, 18084, 18086, 18087, 2537, 2536, 18141, 2548, 2549, 2547, 2630, 2631, 2655, 2632, 2633, 18100, 18101, 2634, 2635, 2921, 18042, 18065, 18152, 2652, 18048, 18102, 18052, 18103, 18053, 2757, 2700, 2687, 2716, 2714, 2742, 2673, 18107, 9549, 2725, 2696, 9591, 9592, 2721, 18111, 2743, 2727, 9542, 18068, 18069, 18070, 2729, 2730, 18169 and 2731
Renal procedures	
Dialysis	9291, 15035, 15036, 15040-15048, 15050, 15051, 332, 493-495, 419, 426, 427, 389, 336, 337
Kidney transplant	771, 773-776, 6221-6224, 6213, 6080, 6081, 6214, 698 and 9489

Comorbidiy	Diagnosis	Medications	Procedures
Cardiovascular	Angina, Ischemic heart disease, transient ischemic attack, hypertension, cerebrovascular disease	ACE inhibitors, Angiotensin II receptor blockers, Diuretics, Beta-Blockers, Calcium channel blockers, Calcium channel blockers, Renin inhibitors, HMG-coenzyme A reductase inhibitors, Fibrates, Lipid agent, Peripheral vasodilator, Anticoagulants, Thrombolytics, Antiplatelet agents, Nitrates	Coronary angiography, Stenting, Echocardiogram and nuclear stress test, Electrocardiogram, Carotid endarterectomy, Bypass surgery, Mechanical clot removal
Bone	Osteoporosis, bone fractures	Bisphosphonate, RANKL inhibitor, Bone formation agent, Hormonal therapy, Supplements of calcium and vitamin D	Cast and bracing, Dexa scans, Surgery for fractures
Renal	Acute glomerulenophretis, nephrotic syndrome, Chronic glomerulonephritis, Nephritis and nephropathy, Acute renal failure, Chronic kidney disease, Renal failure, Dialysis, Renal transplantation, Renal calculi, Cystic diseases of kidney, Diabetic nephropathy, Hypertensive nephropathy	-	Dialysis, Kidney transplant
