# Supplementary material for: Healthcare resource utilization and costs associated with renal, bone and cardiovascular comorbidities among persons living with HIV compared to the general population in Quebec, Canada
Source: PLoS One. 2022 Jul 11;17(7):e0262645. doi: 10.1371/journal.pone.0262645 (PMC9273062; doi:10.1371/journal.pone.0262645)
Supplement: S3 Table — (PDF) [file pone.0262645.s004.pdf]

S3 Table: Health care services utilization and costs for HIV-positive patients with bone comorbidity and for a matched control group of HIV-negative patients with bone comorbidity by age group

Health care services utilization and cost in the 2 years following the bone comorbidity date[a]	HIV-positive patients with bone comorbidity (n=915)		Matched control group HIV-negative patients with bone comorbidity[b] (n=2,745)		p-value[c]
	Mean (SD)	Median (IQR)	Mean (SD)	Median (IQR)	
Patients aged <20 years	(n=21)		(n=63)		
**Number of health care services per patient per year**					
All medical services	8.3 (3.2)	8.0 (3.8)	5.0 (4.1)	4.0 (3.5)	<0.01
Prescription drugs	62.1 (79.9)	34.0 (35.5)	6.0 (11.2)	2.0 (5.5)	<0.01
ART	35.5 (39.1)	23.5 (23.5)	0.0 (0.0)	0.0 (0.0)	<0.01
Other drugs	26.6 (50.0)	4.5 (16.0)	6.0 (11.2)	2.0 (5.5)	0.08
All health care services	70.4 (81.7)	42.0 (35.8)	11.0 (13.6)	6.0 (8.5)	<0.01
Without ART	34.9 (51.9)	13.0 (17.0)	11.0 (13.6)	6.0 (8.5)	0.05
Patients aged between 20-49 years	(n=474)		(n=1,422)		
**Number of health care services per patient per year**					
All medical services	14.8 (20.3)	10.3 (10.0)	7.9 (11.6)	5.5 (7.0)	<0.01
Prescription drugs	178.1 (319.0)	85.3 (132.8)	41.7 (105.8)	8.0 (36.5)	<0.01
ART	45.4 (68.7)	27.0 (21.0)	0.0 (0.0)	0.0 (0.0)	<0.01
Other drugs	132.8 (258.3)	56.5 (111.0)	41.7 (105.8)	8.0 (36.5)	<0.01
All health care services	192.9 (322.0)	98.0 (140.3)	49.6 (109.2)	15.5 (42.0)	<0.01
Without ART	147.5 (261.8)	69.0 (130.1)	49.6 (109.2)	15.5 (42.0)	<0.01
Patients aged between 50-65 years	(n=335)		(n=1,005)		
**Number of health care services per patient per year**					
All medical services	15.6 (20.3)	10.5 (9.0)	9.6 (14.0)	6.5 (8.0)	<0.01
Prescription drugs	166.8 (202.3)	98.5 (111.5)	62.4 (97.9)	34.5 (62.5)	<0.01
ART	35.4 (36.7)	25.0 (25.0)	0.0 (0.0)	0.0 (0.0)	<0.01
Other drugs	131.4 (175.3)	74.0 (104.5)	62.4 (97.9)	34.5 (62.5)	<0.01
All health care services	182.3 (208.9)	116.5 (123.0)	72.1 (101.8)	43.0 (67.8)	<0.01
Without ART	147.0 (182.1)	90.5 (116.5)	72.1 (101.8)	43.0 (67.8)	<0.01
Patients aged >65 years	(n=85)		(n=255)		
**Number of health care services per patient per year**					
All medical services	22.2 (31.7)	13.5 (12.8)	9.8 (11.6)	8.0 (7.0)	<0.01
Prescription drugs	167.6 (156.6)	99.5 (114.3)	68.5 (79.9)	51.5 (50.5)	<0.01
ART	29.6 (35.0)	12.5 (21.3)	0.0 (0.0)	0.0 (0.0)	<0.01
Other drugs	138.1 (133.1)	92.0 (107.3)	68.5 (79.9)	51.5 (50.5)	<0.01
All health care services	189.9 (167.1)	125.5 (128.3)	78.3 (84.5)	59.5 (55.0)	<0.01
Without ART	160.3 (144.7)	113.0 (119.3)	78.3 (84.5)	59.5 (55.0)	<0.01

HIV: human immunodeficiency virus; ICU: intensive care unit; ED: emergency department; ART: antiretroviral treatment; CAN$: Canadian dollar.

[a] Bone comorbidity date was defined by the date of the first medication, the first diagnosis or the first medical procedure related to bone comorbidity in the 2 years following cohort entry. Patients needed to be covered by the RAMQ Drug Insurance Plan in the 2 years following bone comorbidity date to be included in this section of the analysis.

[a] HIV-negative patients with bone comorbidity in the 2 years following cohort entry and matched for age group and gender to HIV-positive patients with bone comorbidity.

[c] p-value for the comparison of HIV-positive patients with bone comorbidity and the matched control group of HIV-negative patients with bone comorbidity from independent t-test for continuous variables.
