# Supplementary material for: Healthcare resource utilization and costs associated with renal, bone and cardiovascular comorbidities among persons living with HIV compared to the general population in Quebec, Canada
Source: PLoS One. 2022 Jul 11;17(7):e0262645. doi: 10.1371/journal.pone.0262645 (PMC9273062; doi:10.1371/journal.pone.0262645)
Supplement: S4 Table — (PDF) [file pone.0262645.s005.pdf]

S4 Table: Health care services utilization and costs for HIV-positive patients with CV comorbidity and for a matched control group of HIV-negative patients with CV comorbidity by age group

Health care services utilization and cost in the 2 years following the CV comorbidity date[a]	HIV-positive patients with CV comorbidity (n=1,498)		Matched control group HIV-negative patients with CV comorbidity[b] (n=4,494)		p-value[c]
	Mean (SD)	Median (IQR)	Mean (SD)	Median (IQR)	
Patients aged <20 years	(n=2)		(n=6)		
**Number of health care services per patient per year**					
All medical services	22.0 (17.7)	22.0 (-)	5.8 (3.1)	5.8 (5.5)	0.42
Prescription drugs	157.8 (161.6)	157.8 (-)	20.5 (33.5)	3.3 (41.9)	0.44
ART	56.3 (50.0)	56.3 (-)	0.0 (0.0)	0.0 (0.0)	0.02
Other drugs	101.5 (111.7)	101.5 (-)	20.5 (33.5)	3.3 (41.9)	0.49
All health care services	179.8 (179.3)	179.8 (-)	26.3 (35.4)	10.5 (45.1)	0.44
Without ART	123.5 (129.4)	123.5 (-)	26.3 (35.4)	10.5 (45.1)	0.48
Patients aged between 20-49 years	(n=684)		(n=2,052)		
**Number of health care services per patient per year**					
All medical services	13.6 (18.1)	9.5 (9.5)	7.1 (8.8)	5.0 (6.5)	<0.01
Prescription drugs	154.7 (225.9)	81.0 (103.3)	49.6 (86.1)	23.0 (45.5)	<0.01
ART	40.2 (40.8)	27.5 (19.0)	0.0 (0.0)	0.0 (0.0)	<0.01
Other drugs	114.6 (195.2)	51.8 (94.5)	49.6 (86.1)	23.0 (45.5)	<0.01
All health care services	168.3 (229.5)	91.8 (113.9)	56.7 (88.9)	29.0 (51.0)	<0.01
Without ART	128.1 (198.9)	62.5 (99.0)	56.7 (88.9)	29.0 (51.0)	<0.01
Patients aged between 50-65 years	(n=620)		(n=1,860)		
**Number of health care services per patient per year**					
All medical services	13.3 (15.1)	10.0 (9.0)	7.2 (6.7)	5.5 (6.5)	<0.01
Prescription drugs	141.5 (176.9)	87.3 (85.4)	55.2 (75.2)	36.5 (47.0)	<0.01
ART	33.4 (33.7)	25.0 (22.0)	0.0 (0.0)	0.0 (0.0)	<0.01
Other drugs	108.1 (151.8)	60.8 (79.3)	55.2 (75.2)	36.5 (47.0)	<0.01
All health care services	154.8 (180.8)	98.5 (87.9)	62.4 (77.2)	43.0 (49.9)	<0.01
Without ART	121.5 (156.1)	73.5 (84.3)	62.4 (77.2)	43.0 (49.9)	<0.01
Patients aged >65 years	(n=192)		(n=576)		
**Number of health care services per patient per year**					
All medical services	17.4 (21.3)	12.8 (11.4)	8.6 (6.1)	8.0 (7.4)	<0.01
Prescription drugs	120.7 (120.8)	81.3 (78.4)	56.6 (56.6)	45.5 (46.0)	<0.01
ART	25.0 (27.1)	12.5 (17.5)	0.0 (0.0)	0.0 (0.0)	<0.01
Other drugs	95.7 (103.0)	66.5 (74.5)	56.6 (56.6)	45.5 (46.0)	<0.01
All health care services	138.1 (129.3)	97.8 (84.3)	65.2 (58.8)	54.3 (48.9)	<0.01
Without ART	113.0 (112.2)	82.8 (83.5)	65.2 (58.8)	54.3 (48.9)	<0.01

HIV: human immunodeficiency virus; ICU: intensive care unit; ED: emergency department; ART: antiretroviral treatment; CAN$: Canadian dollar.

[a] Renal comorbidity date was defined by the date of the first diagnosis or the first medical procedure related to renal comorbidity in the 2 years following cohort entry. Patients needed to be covered by the RAMQ Drug Insurance Plan in the 2 years following renal comorbidity date to be included in this section of the analysis.

[a] HIV-negative patients with renal comorbidity in the 2 years following cohort entry and matched for age group and gender to HIV-positive patients with renal comorbidity.

[c] p-value for the comparison of HIV-positive patients with renal comorbidity and the matched control group of HIV-negative patients with renal comorbidity from independent t-test for continuous variables.
