# Supplementary material for: Healthcare resource utilization and costs associated with renal, bone and cardiovascular comorbidities among persons living with HIV compared to the general population in Quebec, Canada
Source: PLoS One. 2022 Jul 11;17(7):e0262645. doi: 10.1371/journal.pone.0262645 (PMC9273062; doi:10.1371/journal.pone.0262645)
Supplement: S5 Table — (PDF) [file pone.0262645.s006.pdf]

S5 Table: Health care services utilization and costs for HIV-positive patients with renal comorbidity and for a matched control group of HIV-negative patients with renal comorbidity by age group

Health care services utilization and cost in the 2 years following the renal comorbidity date[a]	HIV-positive patients with renal comorbidity (n=191)		Matched control group HIV-negative patients with renal comorbidity[b] (n=573)		p-value[c]
	Mean (SD)	Median (IQR)	Mean (SD)	Median (IQR)	
Patients aged <20 years	(n=1)		(n=3)		
**Number of health care services per patient per year**					
All medical services	35.0 (-)	-	5.3 (3.0)	5.0 (-)	0.01
Prescription drugs	272.0 (-)	-	3.3 (5.8)	0.0 (-)	<0.01
ART	91.5 (-)	-	0.0 (0.0)	0.0 (-)	-
Other drugs	180.5 (-)	-	3.3 (5.8)	0.0 (-)	<0.01
All health care services	307.0 (-)	-	8.7 (6.3)	8.5 (-)	<0.01
Without ART	215.5 (-)	-	8.7 (6.3)	8.5 (-)	<0.01
Patients aged between 20-49 years	(n=76)		(n=228)		
**Number of health care services per patient per year**					
All medical services	26.8 (42.0)	14.0 (9.6)	15.9 (28.1)	9.0 (10.9)	0.04
Prescription drugs	161.4 (138.3)	110.3 (199.4)	62.8 (108.6)	23.0 (81.6)	<0.01
ART	40.1 (35.0)	29.3 (32.5)	0.0 (0.0)	0.0 (0.0)	<0.01
Other drugs	121.3 (122.2)	82.3 (168.8)	62.8 (108.6)	23.0 (81.6)	<0.01
All health care services	188.2 (151.8)	128.0 (204.4)	78.7 (117.8)	32.3 (93.0)	<0.01
Without ART	148.1 (136.9)	103.0 (174.4)	78.7 (117.8)	32.3 (93.0)	<0.01
Patients aged between 50-65 years	(n=79)		(n=237)		
**Number of health care services per patient per year**					
All medical services	26.1 (37.2)	14.5 (17.0)	14.5 (24.6)	8.0 (10.3)	0.01
Prescription drugs	193.2 (246.8)	109.5 (94.0)	68.9 (98.1)	43.0 (75.3)	<0.01
ART	36.3 (38.5)	25.0 (25.0)	0.0 (0.0)	0.0 (0.0)	<0.01
Other drugs	156.9 (216.0)	83.0 (100.0)	68.9 (98.1)	43.0 (75.3)	<0.01
All health care services	219.3 (259.2)	123.0 (174.0)	83.4 (107.2)	53.5 (86.5)	<0.01
Without ART	183.0 (228.7)	105.0 (170.0)	83.4 (107.2)	53.5 (86.5)	<0.01
Patients aged >65 years	(n=35)		(n=105)		
**Number of health care services per patient per year**					
All medical services	33.1 (46.8)	17.5 (19.0)	14.0 (15.7)	11.5 (9.3)	0.02
Prescription drugs	139.5 (166.2)	81.0 (70.5)	86.0 (110.4)	57.0 (74.3)	0.08
ART	23.9 (30.5)	12.0 (14.5)	0.0 (0.0)	0.0 (0.0)	<0.01
Other drugs	115.6 (138.6)	71.5 (80.0)	86.0 (110.4)	57.0 (74.3)	0.20
All health care services	172.6 (190.9)	105.5 (73.0)	100.1 (112.9)	69.5 (77.8)	0.04
Without ART	148.7 (165.1)	97.5 (76.0)	100.1 (112.9)	69.5 (77.8)	0.11

HIV: human immunodeficiency virus; ICU: intensive care unit; ED: emergency department; ART: antiretroviral treatment; CAN$: Canadian dollar.

[a] Renal comorbidity date was defined by the date of the first diagnosis or the first medical procedure related to renal comorbidity in the 2 years following cohort entry. Patients needed to be covered by the RAMQ Drug Insurance Plan in the 2 years following renal comorbidity date to be included in this section of the analysis.

[a] HIV-negative patients with renal comorbidity in the 2 years following cohort entry and matched for age group and gender to HIV-positive patients with renal comorbidity.

[c] p-value for the comparison of HIV-positive patients with renal comorbidity and the matched control group of HIV-negative patients with renal comorbidity from independent t-test for continuous variables.
