# Supplementary material for: Healthcare resource utilization and costs associated with renal, bone and cardiovascular comorbidities among persons living with HIV compared to the general population in Quebec, Canada
Source: PLoS One. 2022 Jul 11;17(7):e0262645. doi: 10.1371/journal.pone.0262645 (PMC9273062; doi:10.1371/journal.pone.0262645)
Supplement: S6 Table — (PDF) [file pone.0262645.s007.pdf]

S6 Table. Mean total healthcare costs for PLHIV with at least 1 comorbidity vs. matched PLHIV without comorbidity

	PLHIV with ≥1 Comorbidity	Matched PLHIV Without Comorbidity†
n	1,336	1,336
**Demographics and Patient Characteristics**		
Mean age, years (SD)	**45.1* (9.5)**	42.3 (10.8)
Charlson comorbidity score, mean (SD)	**3.7* (3.4)**	2.7 (3.1)
Von Korff comorbidity score, mean (SD)	**3.8* (3.2)**	1.4 (1.9)
**Healthcare Utilization**		
**All Medical Services‡**	**12.9* (15.9)**	7.3 (5.5)
Outpatient visits, mean #/pt/yr (SD)	**10.9* (14.8)**	6.3 (4.8)
Hospitalization days, mean # days/pt/yr (SD)	**3.4* (10.9)**	1.0 (4.0)
ED visits, mean #/pt/yr (SD)	**1.3* (3.1)**	0.7 (1.5)
ICU days, mean # days/pt/yr (SD)	**0.2* (1.3)**	<0.1 (0.3)
**Prescription drugs excluding ART, mean #/pt/yr (SD)**	**110.7* (213.5)**	41.8 (122.2)
**Healthcare Costs**		
Total healthcare costs, mean CAD$ (SD)	**$9,184* (16,255)**	$3,366 (6,282)
Difference in mean total healthcare costs/pt/yr between groups, mean CAD$	$5,818	
