# Supplementary material for: Healthcare resource utilization and costs associated with renal, bone and cardiovascular comorbidities among persons living with HIV compared to the general population in Quebec, Canada
Source: PLoS One. 2022 Jul 11;17(7):e0262645. doi: 10.1371/journal.pone.0262645 (PMC9273062; doi:10.1371/journal.pone.0262645)
Supplement: S2 Table — (PDF) [file pone.0262645.s003.pdf]

S2 Table .Health care utilization by age group.

		All Ages		<20 years old		20-49 years old		50-65 years old		>65 years old	
	**Healthcare Utilization**	**PLHIV**	**Control Patients**	**PLHIV**	**Control Patients**	**PLHIV**	**Control Patients**	**PLHIV**	**Control Patients**	**PLHIV**	**Control Patients**
**Any CV Comorbidity‡**	n	1,498	4,494	2	6	684	2052	620	1860	192	576
	**All Medical Services‖**	**14.0* (17.4)**	7.3 (7.7)	22.0 (17.7)	5.8 (3.1)	**13.6* (18.1)**	7.1 (8.8)	**13.3* (15.1)**	7.2 (6.7)	**17.4* (21.3)**	8.6 (6.1)
	Outpatient visits, mean #/pt/yr (SD)	**12.0* (16.4)**	6.3 (6.9)	18.0 (12.7)	4.6 (3.1)	**11.5* (16.9)**	6.0 (8.0)	**11.6* (14.1)**	6.2 (6.0)	**15.3* (20.5)**	7.6 (5.2)
	Hospitalization days, mean # days/pt/yr (SD)	**3.7* (11.8)**	1.3 (6.6)	27.5 (38.2)	2.8 (6.5)	**3.6* (11.3)**	1.4 (6.9)	**3.8* (12.9)**	1.3 (7.1)	**3.5* (8.8)**	1.2 (3.1)
	ED visits, mean #/pt/yr (SD)	**1.2* (2.8)**	0.7 (1.5)	1.5 (2.1)	0.8 (1.1)	**1.4* (3.5)**	0.8 (1.7)	**1.0* (2.0)**	0.6 (1.3)	1.1† (2.5)	0.6 (1.1)
	ICU days, mean # days/pt/yr (SD)	**0.3* (1.4)**	0.1 (0.7)	2.3 (3.2)	0.3 (0.8)	**0.2*** (1.0)	0.1 (0.8)	**0.3* (1.6)**	0.1 (0.6)	0.3 (1.5)	0.2 (0.9)
	**Prescription drugs, mean #/pt/yr (SD)**	**109.5* (168.2)**	52.8 (78.4)	101.5 (111.7)	20.5 33.5)	**114.6* (195.2)**	49.6 (86.1)	**108.1* (151.8)**	55.2 (75.2)	**95.7* (103.0)**	56.6 (56.6)
	**All CV related medical services,¶** mean #/pt/yr (SD)	**1.3* (4.1)**	1.0 (2.7)	n/a	n/a	n/a	n/a	n/a	n/a	n/a	n/a
	**CV prescription drugs,¶** mean #/pt/yr (SD)	**29.5* (41.0)**	22.0 (28.1)	n/a	n/a	n/a	n/a	n/a	n/a	n/a	n/a
**Any Bone Comorbidity‡**	n	915	2,745	21	63	474	1,422	335	1,005	85	255
	**All Medical Services‖**	**15.6* (21.5)**	8.7 (12.5)	**8.3* (3.2)**	5.0 (4.1)	**14.8* (20.3)**	7.9 (11.6)	**15.6* (20.3)**	9.6 (14.0)	**22.2* (31.7)**	9.8 (11.6)
	Outpatient visits, mean #/pt/yr (SD)	**13.2* (20.3)**	7.3 (11.6)	**7.0* (2.8)**	3.9 (3.6)	**12.2* (18.9)**	6.4 (10.5)	**13.5* (19.1)**	8.3 (13.2)	**19.5* (30.9)**	8.9 (11.2)
	Hospitalization days, mean # days/pt/yr (SD)	**4.0* (12.1)**	1.8 (8.1)	**1.2* (0.9)**	0.2 (0.6)	**3.7* (10.1)**	2.0 (9.6)	**4.6* (15.5)**	1.9 (7.0)	**4.2* (8.1)**	1.1 (2.8)
	ED visits, mean #/pt/yr (SD)	**1.6* (3.9)**	1.0 (1.9)	**0.3* (0.6)**	1.0 (1.1)	**1.8* (4.5)**	1.2 (2.1)	1.3† (3.2)	0.9 (1.8)	1.5† (3.5)	0.5 (0.9)
	ICU days, mean # days/pt/yr (SD)	**0.3* (1.7)**	0.1 (0.9)	0.0	0.0	0.2 (1.0)	0.1 (1.1)	0.4† (2.5)	0.1 (0.5)	0.3 (1.0)	0.1 (0.6)
	**Prescription drugs, mean #/pt/yr (SD)**	**130.3* (218.4)**	50.9 (100.3)	26.6 (50.0)	6.0 (11.2)	**132.8* (258.3)**	41.7 (105.8)	**131.4* (175.3)**	62.4 (97.9)	**138.1* (133.1)**	68.5 (79.9)
	**All bone related medical services,****mean #/pt/yr (SD)	**0.7* (2.2)**	1.2 (2.2)	n/a	n/a	n/a	n/a	n/a	n/a	n/a	n/a
	**Bone prescription drugs,****mean #/pt/yr (SD)	**17.1* (27.3)**	6.1 (13.9)	n/a	n/a	n/a	n/a	n/a	n/a	n/a	n/a
**Any Renal Comorbidity‡**	N	191	573	1	3	76	228	79	237	35	105
	**All Medical Services‖**	**27.7* (40.8)**	14.9 (24.7)	**35.0* (-)**	5.3 (3.0)	26.8† (42.0)	15.9 (28.1)	**26.1* (37.2)**	14.5 (24.6)	33.1† (46.8)	14.0 (15.7)
	Outpatient visits, mean #/pt/yr (SD)	**24.9* (40.1)**	12.7 (23.9)	27.5† (-)	4.0 (2.6)	24.2† (41.5)	13.1 (27.2)	23.0† (36.4)	12.6 (23.9)	30.4† (45.9)	12.3 (15.2)
	Hospitalization days, mean # days/pt/yr (SD)	**8.8* (17.9)**	4.3 (14.0)	55.0 (-)	12.7 (15.0)	7.9 (17.4)	4.3 (13.0)	10.4† (20.0)	4.7 (16.6)	5.7 (10.7)	3.3 (8.8)
	ED visits, mean #/pt/yr (SD)	1.6 (2.4)	1.5 (2.6)	3.0† (-)	0.5 (0.5)	1.5 (2.0)	2.0 (3.5)	1.8 (2.9)	1.2 (1.8)	1.2 (1.9)	1.0 (1.4)
	ICU days, mean # days/pt/yr (SD)	0.9 (3.0)	0.4 (1.9)	4.5 (-)	2.7 (4.6)	0.4 (1.2)	0.3 (1.7)	1.2 (3.9)	0.4 (1.3)	0.9 (3.5)	0.7 (3.0)
	**Prescription drugs,¶ mean #/pt/yr (SD)**	**135.3* (169.8)**	69.3 (104.7)	**180.5* (-)**	3.3 (5.8)	**121.3* (122.2)**	62.8 (108.6)	**156.9* (216.0)**	68.9 (98.1)	115.6 (138.6)	86.0 (110.4)
	**All renal related medical services,††** mean #/pt/yr (SD)	**30.5* (85.2)**	10.5 (48.6)	n/a	n/a	n/a	n/a	n/a	n/a	n/a	n/a
	**Renal prescription drugs,††** mean #/pt/yr (SD)	**32.2* (53.4)**	19.9 (34.1)	n/a	n/a	n/a	n/a	n/a	n/a	n/a	n/a
